# Antenatal tetanus, diphtheria, and acellular pertussis (Tdap) immunization and risk of serogroup 19 IPD in children: An indirect cohort study

**DOI:** 10.1080/21645515.2024.2305522

**Published:** 2024-02-08

**Authors:** Melina Thibault, Geneviève Deceuninck, Caroline Quach, Nicholas Brousseau

**Affiliations:** a Department of Epidemiology, Biostatistics, and Occupational Health, McGill University, Montreal, Canada; b Infectious and immune diseases, Centre Hospitalier Universitaire (CHU) de Québec-Université Laval Research Center, Quebec, Canada; c Division of Paediatric Infectious Diseases and Department of Medical Microbiology, Centre Hospitalier Universitaire Sainte-Justine, Montreal, Canada; d Department of Microbiology, Infectious Diseases, and Immunology, University of Montreal, Montreal, Canada; e Biological risks unit, Institut national de santé publique du Québec, Québec, Canada; f Department of Social and Preventive Medicine, Université Laval, Québec, Canada

**Keywords:** immunization, invasive pneumococcal disease, Tdap, interference, vaccination, Blunting

## Abstract

The tetanus-diphtheria-acellular pertussis (Tdap) vaccine has been indicated for pregnant women in Quebec, Canada since 2018. Recent literature suggests maternal Tdap interferes with the pneumococcal vaccine response in children exposed in utero because of maternally transferred anti-diphtheria antibodies, a phenomenon known as blunting. Using an indirect cohort study, we investigated whether maternal Tdap vaccination could alter the protection of PCV vaccines against serotype 19A/F IPD (conjugated to diphtheria toxoid in PCV10). Thirty-seven immunized IPD cases (serotype 19A/F) and 90 immunized IPD controls (non-vaccine serotypes) were analyzed using multivariate logistic regression. Our analyses did not identify antenatal Tdap exposure as a risk factor for IPD in vaccinated children, with and odds ratio close to the null (odds ratio = 0.82, 95%CI = 0.32–2.07). As this study is the first to assess the impact of maternal immunization on pneumococcal disease risk, future investigations involving a larger number of cases should be conducted to confirm or infirm our findings.

## Introduction

The introduction of pneumococcal vaccines in the 2000s led to a substantive decline in the incidence of invasive pneumococcal disease (IPD), a leading cause of childhood mortality.^1^ In the province of Quebec (population roughly 8.6 million), pneumococcal vaccination was recommended for all children <5 years in 2004, using the 7-valent pneumococcal conjugate vaccine (PCV7, Prevnar).^2^ Five years later, the 10-valent vaccine (PCV10, Synflorix) was introduced, followed by the 13-valent vaccine (PCV13, Prevnar13) in 2011.^2^ In 2018, a PCV10-only vaccine schedule replaced PCV13 and in September 2020, a mixed schedule was recommended (i.e. two doses of PCV10 at 2 and 4 months of age and one dose of PCV13 at 1 year).

In Quebec, IPD caused by serotype 19A emerged as a dominant strain among children less than 5 years of age in the PCV7 era, with a maximum of 61 cases reported in 2009, which decreased following the introduction of PCV10 and PCV13 and stagnated at <10 cases per year from 2013 to 2020.^3^ In 2021, a moderate increase was observed mainly for serotype 19A but also for serotype 19F (17 cases of 19A and 5 cases of 19F IPD in children <5 years old), with a stabilization from 2022 onwards (e.g., 16 cases of 19A and one case of 19F IPD case in 2022).

Tetanus, diphtheria, and acellular pertussis (Tdap) vaccination has been recommended for pregnant persons in Quebec since May 2018. The passive transfer of maternal antibodies to the fetus in utero allows for protection against infectious diseases prior to the infant primary vaccination series.^4^ However, current research suggests antenatal Tdap could lead to lower PCV infant immune response whereby maternally transferred anti-diphtheria toxoid (DT) antibodies target the diphtheria toxin mutants (CRM197) or DT carrier proteins in pneumococcal vaccines.^5^ Maternal Tdap has been associated with decreased infant antibody responses to serotypes 19A/19F for PCV10, and for all serotypes for PCV13.^5–12^ This is likely because all serotypes in PCV13 are conjugated to diphtheria toxin mutant CRM197, whereas, only serotype 19F is conjugated to diphtheria toxoid in PCV10.^5^ Although immunological interference is well documented, no studies to date have indicated a decreased vaccine effectiveness in children.

In Quebec, the temporary rise in serotype 19A and 19F IPD cases coincided temporally with the introduction of maternal Tdap immunization. With data collected from pediatric IPD cases, this study aimed to investigate whether maternal Tdap vaccination could alter the PCV vaccines’ protection against serotype 19A/F IPD in immunized children.

## Materials and methods

### Study design

We used an indirect cohort design (‘Broome’ method), a modified case-control methodology, where cases were IPD patients with 19A/F serotype and controls were IPD patients with non-vaccine serotypes (nor vaccine-related serotypes). As the objective was to evaluate if maternal Tdap vaccination modified the infant protection through PCV, the analysis was restricted to vaccinated infants.

### Population

We looked at all serotyped IPD cases in vaccinated children aged 2–59 months at the time of diagnosis, born after January 1^st^, 2018 (to capture mothers targeted by antenatal immunization). These cases were confirmed by culture or polymerase chain reaction (PCR) from a normally sterile site, notified to regional health authorities from 2018 until May 29, 2023, for whom individual and maternal immunization status could be ascertained, with at least one dose of PCV10 or PCV13. Patients with IPD from vaccine serotypes other than 19A/F (1, 3, 4, 5, 6A, 6B, 7F, 9V, 14, 18C, 23F) or vaccine-related serotypes (e.g., those of the same serogroup but not of the same serotype as PCV13 such as 18A/F, 7A, 9A, and 6B/C) were excluded from the study. Any PCV dose given ≥10 days before specimen collection date was included.

Infants were considered exposed to antenatal Tdap vaccination if their mother was vaccinated between 3 and 36 weeks before delivery. Participants were excluded if maternal vaccination occurred <3 weeks or within 37–140 weeks before delivery. Sensitivity analyses restricted patients to those who received a PCV10-only vaccine schedule.

### Data collection

Data for this study was gathered from several sources under an ongoing surveillance mandate from the Quebec Ministry of Health and Social Services. IPD is a notifiable disease under this provincial mandate. Once a case was confirmed, the medical record was reviewed and disease-related characteristics were documented (vaccination status, risk factors, disease severity, etc.). Maternal immunization status was ascertained through the Provincial Vaccination Registry Database, where all immunizations have to be recorded by law.^13^

### Statistical analyses

Descriptive analyses, including univariate logistic regression, were used to examine covariates. As the number of PCV doses received and the risk of disease vary by age, regression models were adjusted by age category. The main analysis was a multivariable logistic regression to calculate the odds ratio (with 95% confidence intervals [CI]) of maternal Tdap vaccination (compared to no maternal vaccination) among cases and controls. Results were adjusted for age category (2–11, 12–23, 24–59 months), calendar year (2018–2019, 2020, 2021, 2022, 2023), and type of vaccine received (PCV10-only, PCV13-only, mixed schedule). Two-sided *p* value of < .05 was considered statistically significant. All analyses were performed using R 4.2.1 software.

### Ethics

This study was part of a mandate from the Quebec Ministry of Health and Social Services and authorization from an ethics research board was therefore not necessary.

## Results

**Figure 1.**
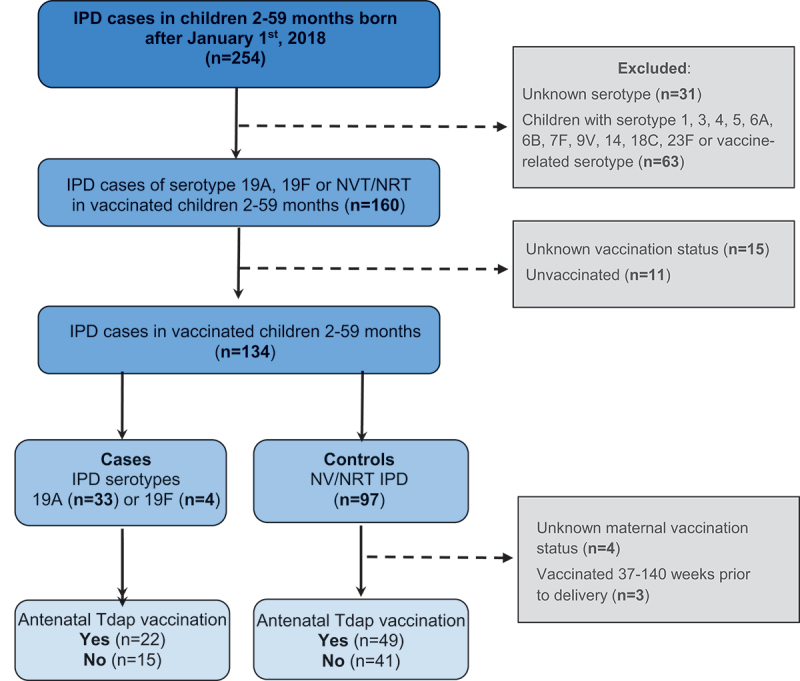
Flowchart describing cases and controls included in the study.

Of the 254 IPD cases identified through the provincial registry of notifiable diseases during the study period, 134 pediatric cases met the study criteria, but 7 were excluded because either the mother’s immunization status was unknown (*n* = 4), or the mother received their Tdap 37–140 weeks prior to delivery (*n* = 3). Thirty-seven patients were considered cases: four serotype 19F and thirty-three serotype 19A IPD (Figure 1). Among the 90 controls, serotype 22F (24.4%), 15B (15.6%), 15A (8.9%), and 33F (8.9%) were the most prevalent.

*IPD, invasive pneumococcal disease; NV/NRT, non-vaccine/non-vaccine-related serotypes; Tdap, tetanus, diphtheria, and acellular pertussis vaccine.*

Of the 254 IPD cases identified, 134 were eligible. Among them, 7 individuals were excluded (three had parents who received their Tdap <2 years preceding their pregnancy and for four persons, we were unable to ascertain maternal vaccination status). Of the 127 IPD patients, 37 were considered cases: four serotype 19F and thirty-three serotype 19A IPD.

In cases, 22 (59.5%) patients were considered exposed to antenatal vaccination and 15 (40.5%) were unexposed (mothers were unvaccinated, received their Tdap more than 2 years before pregnancy, or received Tdap after delivery) (Table 1). Among controls, 54.4% were exposed and 45.6% were unexposed. Eight infants were premature, among them, three mothers received their Tdap, in all cases administered 5–8 weeks prior to delivery.

**Table 1.** Summary of sociodemographic variables collected for all IPD patients.

Characteristics	Cases (N = 37)	Controls (N = 90)	Total (N = 127)
*Exposure to Antenatal Vaccination*			
Yes	22 (59.5%)	49 (54.4%)	71 (55.9%)
No	15 (40.5%)	41 (45.6%)	56 (44.1%)
*Age Category*			
<1 year	21 (56.8%)	31 (34.4%)	52 (40.9%)
12–23 months	4 (10.8%)	41 (45.6%)	45 (35.4%)
≥2 years	12 (32.4%)	18 (20.0%)	30 (23.6%)
*Year of Diagnosis*			
2018	0 (0.0%)	1 (1.1%)	1 (0.8%)
2019	1 (2.7%)	13 (14.4%)	14 (11.0%)
2020	5 (13.5%)	19 (21.1%)	24 (18.9%)
2021	14 (37.8%)	26 (28.9%)	40 (31.5%)
2022	13 (35.1%)	22 (24.4%)	35 (27.6%)
2023	4 (10.8%)	9 (10.0%)	13 (10.2%)
*Vaccine Schedule*			
Mixed	3 (8.1%)	36 (40.0%)	39 (30.7%)
Only PCV10	33 (89.2%)	49 (54.4%)	82 (64.6%)
Only PCV13	1 (2.7%)	5 (5.6%)	6 (4.7%)
*Total PCV Doses Received*			
1	6 (16.2%)	5 (5.6%)	11 (8.7%)
2	21 (56.8%)	30 (33.3%)	51 (40.2%)
3	10 (27.0%)	52 (57.8%)	62 (48.8%)
4	0 (0.0%)	3 (3.3%)	3 (2.4%)

Most infections occurred in 2021 and 2022, but the proportion was higher for cases (72.9%) than controls (54.8%) (Table 1). Although the mean age for cases (18.2 months) and controls (18.0 months) was not statistically different, cases experienced a bimodal distribution (56.8% of IPDs occurring in those <1 year, 10.8% in 12–23 months, and 32.4% in ≥24 months children). Controls showed a more equal distribution in age categories <1 year (34.4%) and 12–23 months (45.6%). Relatedly, a higher proportion of cases than controls received ≤2 PCV doses (73.0% vs. 38.9%, respectively). Vaccine schedule distributions varied between groups, with 89.2% of cases receiving only PCV10 and 8.1% a mixed schedule of PCV10 and PCV13, compared to 54.8% and 40.0%, respectively, for controls.

Using multivariable logistic regression, the odds of 19A/F IPD cases having been exposed to antenatal vaccination was 0.82 (95% CI, 0.32–2.07), after adjusting for calendar year, age, and vaccine schedule. Ad-hoc sub-analyses among patients with a PCV-10-only vaccine schedule showed an odds of exposure to antenatal vaccination of 0.78 (95% CI, 0.28–2.11), after adjusting for calendar year and age. Further analyses among patients <24 months of age showed an odds of having been exposed to antenatal vaccination of 0.81 (95% CI, 0.27–2.47), after adjusting for calendar year and vaccine schedule. Due to the small sample size, no analyses restricted to a PCV13-only or mixed vaccine schedule were possible.

## Discussion

In this exploratory indirect cohort study, our analyses did not identify antenatal Tdap exposure as a risk factor for IPD in vaccinated children, with and odds ratio close to the null (odds ratio = 1). Moreover, when analyses were restricted to the critical period (<24 months), and/or to patients with a PCV10-only schedule, the estimated association was similarly close to the null.

To our knowledge, the present exploratory study is the first to assess the impact of maternal immunization on pneumococcal disease risk. Previous studies assessed the impact of antenatal immunization in vaccinated children (>6 months of age), noting lower anti-capsular pneumococcal IgG levels.^5–10,14,15^ While the latter suggests a baseline immunological interference, there is no strong indication this has a clinical impact on vaccine effectiveness (VE). Moreover, it appears that immune interference is attenuated or disappears after the 12-month booster dose.^9,11^

It is possible the moderate rise in Quebec IPD cases, occurring mostly in 2021 among infants, was related to a major RSV outbreak, facilitated by the 2018 to 2020 province-wide PCV10-only vaccine schedule.^16^ The burden of disease from RSV is greatest in children <1 year,^17^ favoring secondary pneumococcal infections, leading to IPD. Moreover, the World Health Organization evaluated PCV10-only schedules as generating suboptimal indirect protection against serotype 19A.^18^ The significant proportion of serotype 19A IPD observed in Quebec is a trend similarly observed in Belgium and New Zealand, following the switch from PCV13 to PCV10 in the countries’ immunization programs.^18,19^ However, there is insufficient evidence to determine if one of these two vaccines has a higher impact on overall IPD burden (when combining vaccine-type and non-vaccine-type disease burden), due to an increase of non-vaccine serotypes after PCV introduction, i.e. serotype replacement.^20^

Serotype-based pneumococcal vaccines provide only serotype-specific immunity, thus serotype replacement remains a threat, driving the development of non-serotype specific vaccines that could protect against both IPD and non-IPD infections.^21^ Non-serotype specific vaccines would help to improve the effectiveness and impact of pneumococcal vaccines. As a result, pneumococcal protein vaccines are being investigated in both preclinical and clinical settings.^22^ These developing next-generation vaccines, including those targeting biofilm formation, are one avenue of interest also often explored for *Staphylococcus aureus* and *Staphylococcus epidermidis* .^23^ Given the distinct characteristics of these bacterial genera, gram-positive putative vaccines should consider factors influencing virulence profiles, including co-occurrence in the infection environment and the complex mechanistic interplay of these opportunistic pathogens at the level of the respiratory microbiome.^24,25^

This study had a few limitations, the main one being the small sample size resulting in less precise estimates, constrained variables adjusted for, and limited possible stratifications. Indeed, covariate distributions were less obvious in this small sample size, but differences in baseline characteristics between groups could be discerned; serogroup 19 cases were on average younger and proportionally had received less PCV doses. Larger studies with more precise estimates are required to determine if antenatal Tdap exposure is a risk factor for pneumococcal disease in vaccinated children. Surveillance systems in countries such as the United States and England, covering a larger population, could be used to develop a similar study with a larger sample size. Secondly, the Broome method assumes that the risk of non-vaccine-type (NVT) IPD is equal for both immunized and unimmunized patients, if this assumption does not hold true, there is a potential for bias.^26^ However, the study included only PCV-immunized children, which theoretically should negligibly affect the risk of NVT IPD.^26^

## Conclusion

While the immunological blunting of pneumococcal infant immune responses following exposure to antenatal Tdap vaccination is established in literature, our study was unable to conclude that it was a significant modifier of PCV protection in vaccinated children. Our exploratory study was limited by its small sample size and larger epidemiological studies should be conducted to confirm or infirm our findings.

## Data Availability

Due to the nature of the research and to ethnical and legal restrictions, supporting data is not available.
